# Fluorinated methacrylamide chitosan hydrogel dressings enhance healing in an acute porcine wound model

**DOI:** 10.1371/journal.pone.0203371

**Published:** 2018-09-05

**Authors:** Pritam S. Patil, M. Michelle Evancho-Chapman, Hang Li, He Huang, Richard L. George, Leah P. Shriver, Nic D. Leipzig

**Affiliations:** 1 Department of Chemical and Biomolecular Engineering, University of Akron, Akron, Ohio, United States of America; 2 Department of Surgery, Summa Health Systems–Akron Campus, Akron, Ohio, United States of America; 3 Department of Chemistry, University of Akron, Akron, Ohio, United States of America; Universite de Technologie de Compiegne, FRANCE

## Abstract

Wound healing involves multiple interrelated processes required to lead to successful healing outcomes. Phagocytosis, inflammation, cell proliferation, angiogenesis, energy production, and collagen synthesis are all directly or indirectly dependent on oxygen. Along with other critical factors, such as nutrition and comorbidities, availability of oxygen is a key determinant of healing success. Previously, we have presented a novel oxygenated hydrogel material that can be made into dressings for continuous localized oxygen delivery to wounds. In this study, an acute porcine wound model was used to test the healing benefits of these oxygenated MACF (MACF + O_2_) hydrogel dressings compared to controls, which included commercial Derma-Gel^TM^ hydrogel dressings. Wound closure and histological analyses were performed to assess re-epithelialization, collagen synthesis, angiogenesis, and keratinocyte maturation. Results from these assays revealed that wounds treated with MACF + O_2_ hydrogel dressings closed faster as compared to Derma-Gel (*p*<0.05). Targeted metabolomics via liquid chromatography separation and mass spectrometric detection (LC-MS/MS) and a biochemical assay determined the concentration of hydroxyproline in wound samples at days 14 and 21, showing that MACF + O_2_ hydrogel dressings improved wound healing via an upregulated collagen synthesis pathway as compared to Derma-Gel (*p*<0.05). Histological evidence showed that MACF + O_2_ hydrogel dressings improve new blood vessel formation and keratinocyte maturation over all other treatments.

## Introduction

Hydrogels are prepared from synthetic or naturally derived polymers to recapitulate aspects of the native extracellular matrix (ECM) [1]. Hydrogels have enhanced relevance in wound healing treatments since wounds maintained at beneficial moisture levels heal faster [2]. while providing other benefits including providing a substrate for enhanced cell migration, an artificial ECM, and a substrate for bioactive agent release [3]. Several biomaterials such as chitosan, hyaluronic acid (HA), collagen, fibrin, alginate, poly(lactic-co-glycolic acid), polyethylene glycol, their derivatives, and various combinations have been used to formulate hydrogels for wound healing [3,4].

In addition, oxygen plays a significant role in both acute and chronic wound healing, and growing evidence shows that tissue oxygenation level is a key determinant of dermal healing responses [5]. Oxygen treatment has been demonstrated to promote wound healing by enhancing metabolism, ECM synthesis, and neovascularization while limiting antimicrobial activity [6]. Thus, wound oxygenation should be an important consideration for wound therapies [7]. Despite the benefits of supplemental oxygen, current oxygen delivery therapies are intermittent, inconvenient for the patient, and require access to expensive and specialized equipment. Therefore, there is a significant need for a simple wound care dressing able to provide regenerative levels of oxygen to supplement, or possibly supplant current therapies. To provide supplemental oxygen to wounds, researchers have developed several ways such as topical oxygen therapy, oxygen gas flow devices, and oxygenating dressings [8]. Here, we evaluate a unique hydrogel dressing, developed and characterized previously [9], that has the potential to provide uniform and tunable oxygen delivery to a wound.

Our novel technology (methacrylated fluorinated chitosan, MACF) uniquely allows for the creation of sheet hydrogels that provide sustained oxygenation for up to five days at significant oxygen partial pressures [9]. MACF has the innate ability to sequester oxygen from the atmosphere and act as reservoir until exposed to low oxygen environment while providing antioxidant benefits [10]. MACF hydrogel dressings can also be oxygenated until saturation for longer oxygen release. Thus, MACF potentially marries the benefits of oxygen treatment with the advantages of hydrogel dressings (e.g., increased angiogenesis, enhanced autolytic debridement, and increased re-epithelialization). Derma-Gel™, a commercial dressing used for comparison in this study, is an absorbent hydrogel wound dressing containing 65% glycerin. Derma-Gel has been used in treatment of pressure injuries and partial and full thickness acute wounds [11,12]. It helps create the moist environment by locking the moisture within the hydrogel [13]. However, synthetic hydrogels, like Derma-Gel, do not necessarily mimic important ECM properties. Conversely, the chitosan backbone of MACF is similar to the native ECM constituent HA [14,15].

The primary goal of this study was to determine if MACF + O_2_ applied to acute porcine wounds demonstrated improved healing responses. We assessed a commercially available and clinically relevant Derma-Gel hydrogel, MACF, MACF + O_2_ (saturated with oxygen at application) all covered with a non-hydrogel foam dressing and a non-hydrogel control to elucidate the contributions of MACF to wound healing with or without the addition of above atmospheric O_2_.

## Materials and methods

### Oxygenated methacrylated fluorinated chitosan hydrogel dressing makeup

Methacrylated fluorinated chitosan hydrogel dressings were created and characterized as previously described [9]. MACF hydrogel dressings were prepared in sheets with the dimensions of 2.5 cm × 2.5 cm × 0.5 cm. MACF + O_2_ were prepared by saturating MACF hydrogel dressings immersed in phosphate buffered saline (PBS) buffer with 100% pure oxygen for 30 minutes just before application.

### Animal care and housing

Prior to surgery, the swine were acclimated for a minimum of 10 days and a health check by the veterinarian was performed weekly. All swine were given a preventative antibiotic consisting of Augmentin (Clavamox) 10mg/kg by mouth twice a day for seven days before survival surgery. On weekends the total daily dose was given once per day. Before conducting any animal testing, the Institutional Animal Care and Use Committee (IACUC) at the Northeast Ohio Medical University, Rootstown, Ohio approved all experiments. Animals were housed in individual cages in the same room to maintain social interactions. For these studies a female Yorkshire mix swine (Shoup, LLC, Wooster, OH, USA) was used [16]. Pigs were anesthetized and monitored throughout the procedure to maintain a surgical-plane of anesthesia.

### Acute porcine wound model

**Fig 1:**
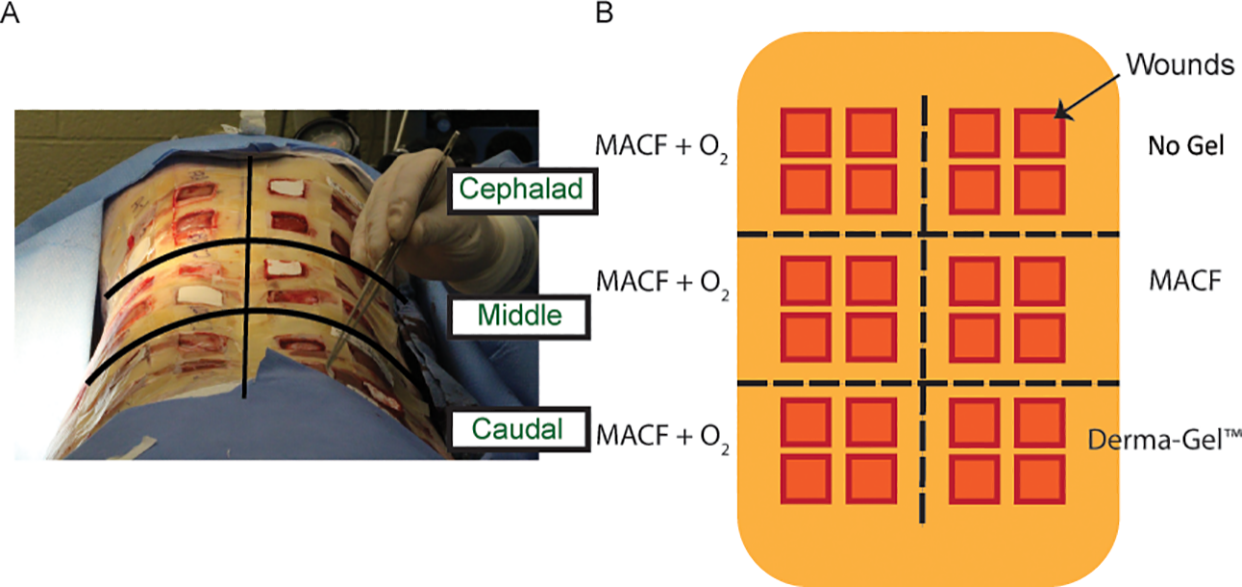
A) Wound arrangement and B) treatment assignment on the back of pigs.

A 2.5 cm × 2.5 cm template was used to create 12 full-thickness wounds each on the right and the left side of the back using a #15 surgical blade to yield 24 wounds per animal (Fig 1) [17]. The study was performed in the Comparative Medicine Unit located on the Northeastern Ohio Medical University campus (NEOMED, USDA 31-R-0092, OLAW A3474-01 and AAALAC accredited) in Rootstown, Ohio. The animal study was conducted according to USDA, OLAW and AAALAC guidelines consistent with the “Guide for the Care and Use of Laboratory Animals”. The NEOMED IACUC approved protocol 14–036 for this was used for this template. Other researchers have previously used 24 wounds in full thickness and split-thickness porcine wound healing [4–6]. A slow-releasing analgesia, Buprenorphine SR (0.1 mg/kg/dose) was administered just prior to the start of surgery. Pain levels were assessed twice daily throughout the study period, and additional analgesia was available as needed, but was not required. Samples from three animals were collected for analysis. Since regional differences are prominent in wound healing [18], wounds were divided into three transverse groups of 8 wounds each to generate the divisions of cephalad, middle, and caudal regions.

### Treatment groups

MACF + O_2_ dressings were applied to all wounds on either the right or the left side in all three regions; thus, 12 wounds per swine were treated with MACF + O_2_. On the corresponding opposite side in each region, comparison treatments of “No Gel” (i.e., no primary dressing), MACF (without oxygen), or Derma-Gel were placed on all 4 wounds in the respective region (Fig 1). The grouped contralateral design allowed for regional skin differences to be accounted for in the analysis. Random assignment within each region was not possible as effect of oxygenation from MACF dressings could possibly confound outcomes of other control treatments. All wounds were covered with secondary non-adherent Allevyn® foam dressings regardless of the primary treatment. The foam was used to hold hydrogel treatments in place and maintain a moist environment with the addition of 1 mL sterile saline injected into the foam dressing at each dressing change. Finally, all dressings were covered by Tegaderm™ and a custom shoulder-to-rump lycra-spandex jacket for protection of dressings [19].

### Dressing changes and wound closure measurements

At post-operative wound days 2, 4, 9, 11, 14, 17, and 21, under general anesthesia, a dressing change was performed, applying a new dressing from the same treatment after gently cleansing the wound bed with sterile saline. At each dressing change, wound images were taken with a digital camera with EF 100 mm macro lens (Canon EOS Rebel SL1, Tokyo, Japan) as well as with a Silhouette® wound imaging system (ARANZ Medical, Christchurch, NZ) to allow for semi-automated quantification of wound area and perimeter for Gilman parameter analysis [17]:

$$Gilman\;Parameter=\frac{\Delta S}{\frac{P_{0}+P_{n}}{2}}$$

where ΔS is the change in wound area from day 0, and P_0_ and P_n_ are the wound perimeters on day 0 and day *n* respectively, with *n* = 2, 4, 9, 11, 14, 17, 21. A higher Gilman parameter signifies greater closure of the wound. The Gilman parameter has a unit of length and is expressed in cm.

### Biopsies for metabolomic, biochemical, histologic, and immunohistochemistry analyses

On post-surgery day 14, a 3 mm biopsy punch was used to harvest wound tissue samples from the lower left or right of the wound, that were fixed for histology and immunohistochemistry (IHC) analyses. Further, biopsies were taken carefully making sure that it did not affect other analyses. On post-surgery days 14 and 21, a 2 mm biopsy punch was used to harvest wound tissue samples from the near centerline of the wound, which were snap frozen and stored in a -80°C freezer for LC-MS/MS based metabolomics analyses. On post-surgery day 21, the animals were sacrificed and the whole wound tissues were excised and fixed for histology, biochemical analysis and IHC analyses.

### Free hydroxyproline content in wound tissue

The amount of free hydroxyproline was quantified using tandem mass spectrometry (LC-MS/MS) in wound tissues similar to our recent rat study [20]. Metabolite extraction was performed with a modified Bligh Dyer method [21]. Extracted metabolites from the aqueous phase were dried in a CentriVap Concentrator (Labconco, Kansas city, MO, USA) and then stored at -80°C until analysis. Protein pellets were used to normalize extracted metabolite quantity by using a bicinchoninic acid assay (G-Biosciences, St. Louis. MO, USA). A hydroxyproline (Sigma-Aldrich, St. Louis, MO, USA) standard curve was obtained by running hydroxyproline solution at 1 × 10^−3^, 1 × 10^−4^, 1 × 10^−5^, 1 × 10^−6^, 1 × 10^−7^, 1 × 10^−8^, 1 × 10^−9^ M concentrations. For hydroxyproline detection, hydrophilic interaction liquid chromatography was performed on a Micro200 LC (Eksigent, Redwood, CA, USA) with a Luna NH_2_ column (3 μ, 100Å, 150mm by 1.0mm, Phenomenex, Torrance, CA, USA). Samples were analyzed on a 5600+ TripleTOF Mass Spectrometer (AB SCIEX, Framingham, MA, USA) and (132.10 → 86.09) m/z transition was used for hydroxyproline detection.

### Biochemical analysis: Total hydroxyproline content in wound tissue

A hydroxyproline assay was used to determine the total hydroxyproline content in each wound tissue sample, corresponding to the total collagen present in tissue sample, as previously published [22,23]. This offered a confirmatory assay to LC-MS/MS analysis of free hydroxyproline and further reveal insights on incorporation of free hydroxyproline in collagen synthesis. This assay was performed on hydrolyzed wound tissue samples using a hydroxyproline assay kit (Cell Biolabs Inc., San Diego, CA, USA). Wound tissue samples were acid hydrolyzed in 6 N HCl at 100°C for 3–4 hours using the hydroxyproline assay kit protocol [24].

### Histology

Histology was performed to visualize and assess healing responses directly. Samples were first paraffin embedded, then sectioned at 12 μm. Hematoxylin and eosin (H&E) staining was performed using the manufacturer’s protocol (EMD Millipore, Billerica, MA, USA). Picrosirius Red (Polysciences Inc, Warminster, PA, USA) was used on the second set of sections to visualize collagen fibers along with a confocal laser scanning microscope (Fluoview FV1000, Olympus, Tokyo, Japan) using optimized settings for FITC (488 nm) and Texas Red (559 nm). Image processing such as directionality analysis and color thresholding was performed in Image J software (National Institutes of Health, Bethesda, MD, USA) to facilitate measurement of collagen area and collagen fiber dispersion/fiber alignment. Additional sections were used for immunostaining using von Willebrand factor (vWF) for neovascularization, and cytokeratin I K17 and cytokeratin II for maturation of keratinocytes. A mouse primary antibodies for anti-vWF (ab6994) (Abcam, Cambridge, UK), Cytokeratin I K17 (Avivasysbio, San Diego, CA, USA), and Cytokeratin II (ImmuQuest, Seamer, North Yorkshire, UK) were used at 1:500 dilution and incubated with sections at 4°C. Endogenous peroxidase activity was inactivated with 3% hydrogen peroxide to reduce non-specific secondary antibody binding. Sections were incubated with horseradish peroxidase-conjugated secondary antibody goat-anti-rabbit (ab6721; Abcam), which was used at 1:500 dilution for 2 hours for vWF and K17 primary. For cytokeratin II, donkey-anti-mouse (Millipore, Temecula, CA, USA), was used at 1:500 dilution for 2 hours. A 3, 3′ diaminobenzidine staining kit (Sigma-Aldrich) was used to develop peroxidase staining. The sections were counterstained with hematoxylin. All slides were imaged on an optical microscope (CKX-41 with DP21 camera, Olympus) and ImageJ software was used for image analyses [25]. H&E images were used to measure the length of epithelial tongues by image analysis using ImageJ software. The length of epithelial tongue was measured from the edge of the wound bed to the tip of the tongue on either side. H&E histology was also used to analyze circulating red blood cells (RBCs) in newly formed capillaries. Images were taken at the center of each wound bed just below the epithelial layer. Further vWF IHC was performed to confirm findings from H&E histology.

### Statistics

The generalized linear model (GLM) repeated measures for analysis of variance (ANOVA) in SAS software (SAS institute, Cary, North Carolina, USA) was used with time as a repeated measure for wounds analyzed over a time-course (e.g., for wound closure). For results from assays with only one or two-time points, ANOVA with *post hoc* analysis was used with an alpha of 0.05. The effect of regions was tested to determine if they should be included as a factor before all statistical analyses.

## Results

### Wound imaging: Wound closure

**Fig 2:**
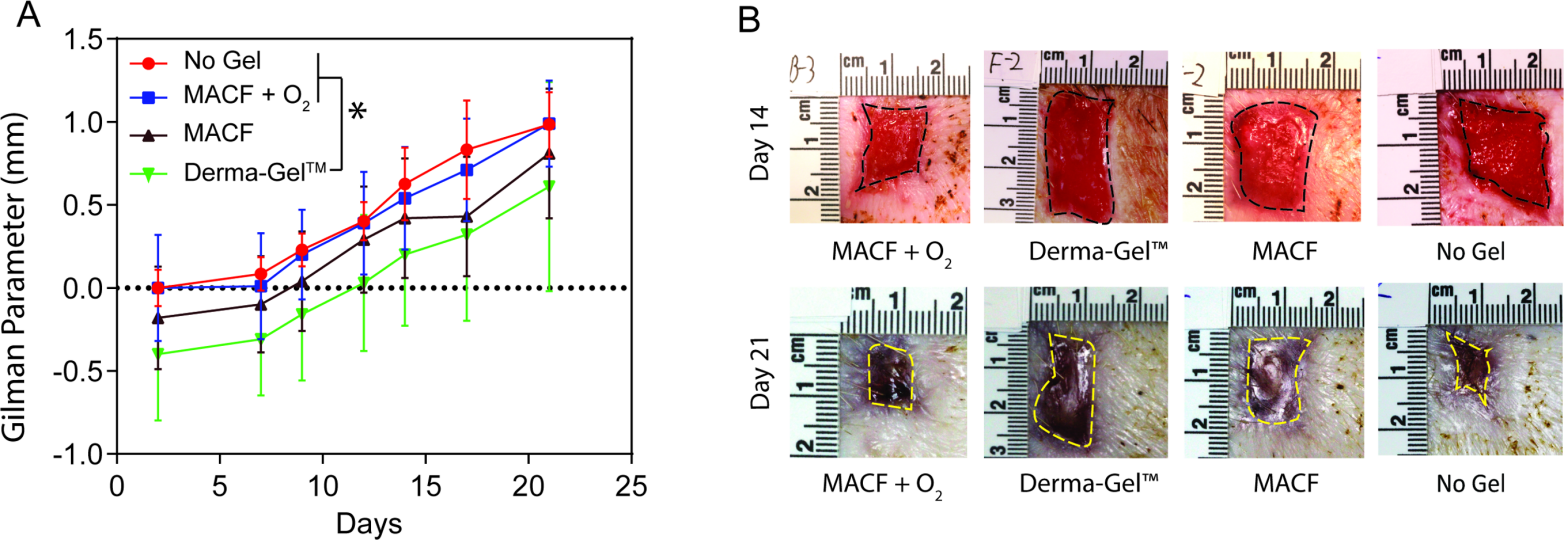
Wound closure shows significant benefits of hydrogel treatment over comparison dressing. A) Gilman parameter analysis. Letters denotes significance by GLM for ANOVA at each time point used as a repeated measure, (*p* < 0.05). Mean+/-SD, *n* = 3 (pigs), s = 4 (wounds/region) wounds for all groups. B) Wound pictures at Day 14 and Day 21.

As MACF + O_2_ dressings were applied in all the regions, regional differences were first analyzed using one-way ANOVA on MACF + O_2_ treated wounds and the test revealed no statistical difference among regions for wound closure analysis (p > 0.05). Hence, region was not considered in this statistical analysis and all MACF + O_2_ Gilman parameter data from 36 wounds (n = 3 (pigs), s = 4 (wounds/region)) was combined and compared against other treatments 12 wounds (n = 3 (pigs), s = 4 (wounds/region)) using GLM repeated measures ANOVA. Results (Fig 2) revealed that MACF + O_2_ dressings presented similar Gilman parameters as compared to No Gel and MACF (p > 0.05). However, MACF + O_2_ significantly outperformed Derma-Gel dressings (*p* < 0.05).

### LC-MS/MS based metabolomics: Free hydroxyproline content in wound tissue

**Fig 3:**
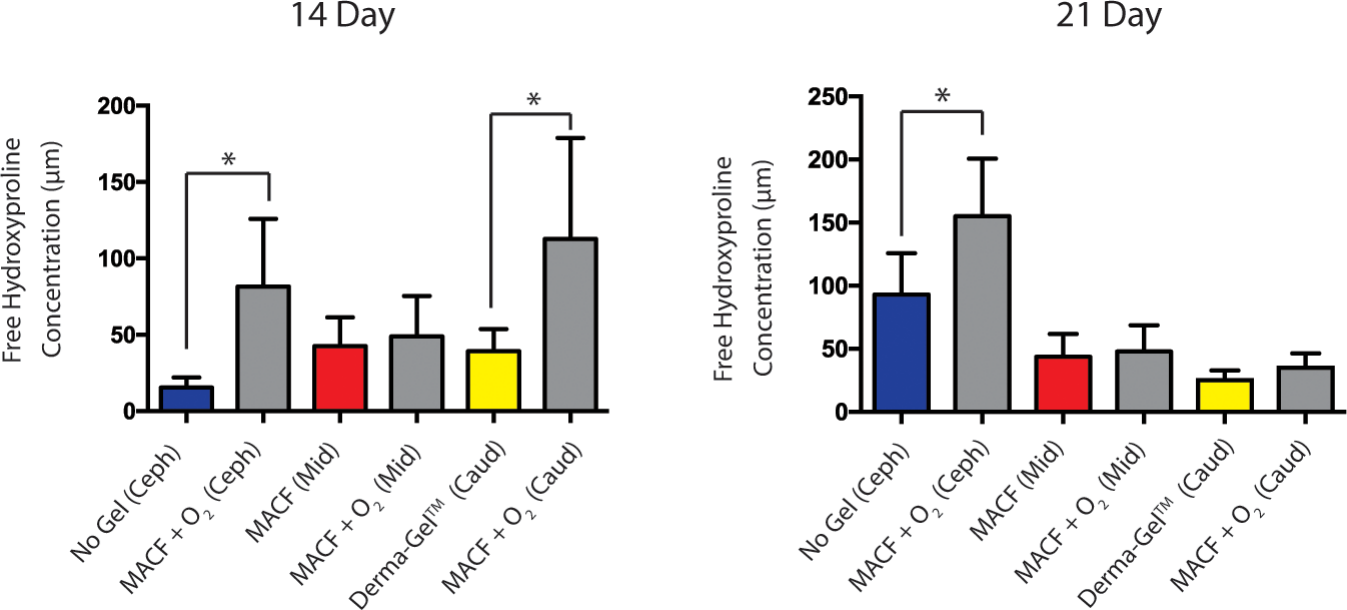
Free hydroxyproline concentration changes in wound samples measured by LC-MS/MS analysis. Regions are denoted as Cephalad (Ceph), middle (Mid), and Caudal (Caud). * denotes significance by one factor ANOVA with Tukey’s post hoc, (*p* < 0.05). Mean+/-SD, *n* = 3 (pigs), s = 2 (wounds/region).

To better probe underlying molecular mechanisms of wound healing responses to MACF + O_2_ dressings and controls, wound samples were analyzed for free hydroxyproline which is utilized in collagen synthesis during wound healing [20]. Statistically significant differences in hydroxyproline levels were found in regions when MACF + O_2_ was compared among regions (*p* < 0.05). Therefore, subsequent statistical analyses were performed to compare this metabolite within the regions with n = 3 (pigs) and s = 2 (wounds/region). Results revealed that free hydroxyproline concentration in MACF + O_2_ treated wounds was significantly higher than No Gel treated wounds (*p* < 0.05) (Fig 3) on both day 14 and day 21. Similarly, MACF + O_2_ treated wounds showed significantly higher concentrations of free hydroxyproline compared to Derma-Gel treated wounds (*p* < 0.05), but, only on day 1. On day 21, a similar trend was observed, but differences were not significant (*p* = 0.11). Interestingly, free hydroxyproline concentration in MACF + O_2_ treated wounds in comparison to Derma-Gel treated wounds dropped from day 14 to day 21. However, MACF + O_2_ treated wounds and MACF treated wounds showed similar trends between day 14 and 21, but showed no significant difference (*p* = 0.64 and 0.71 respectively).

### Biochemical analysis: Total hydroxyproline content in wound tissue

**Fig 4:**
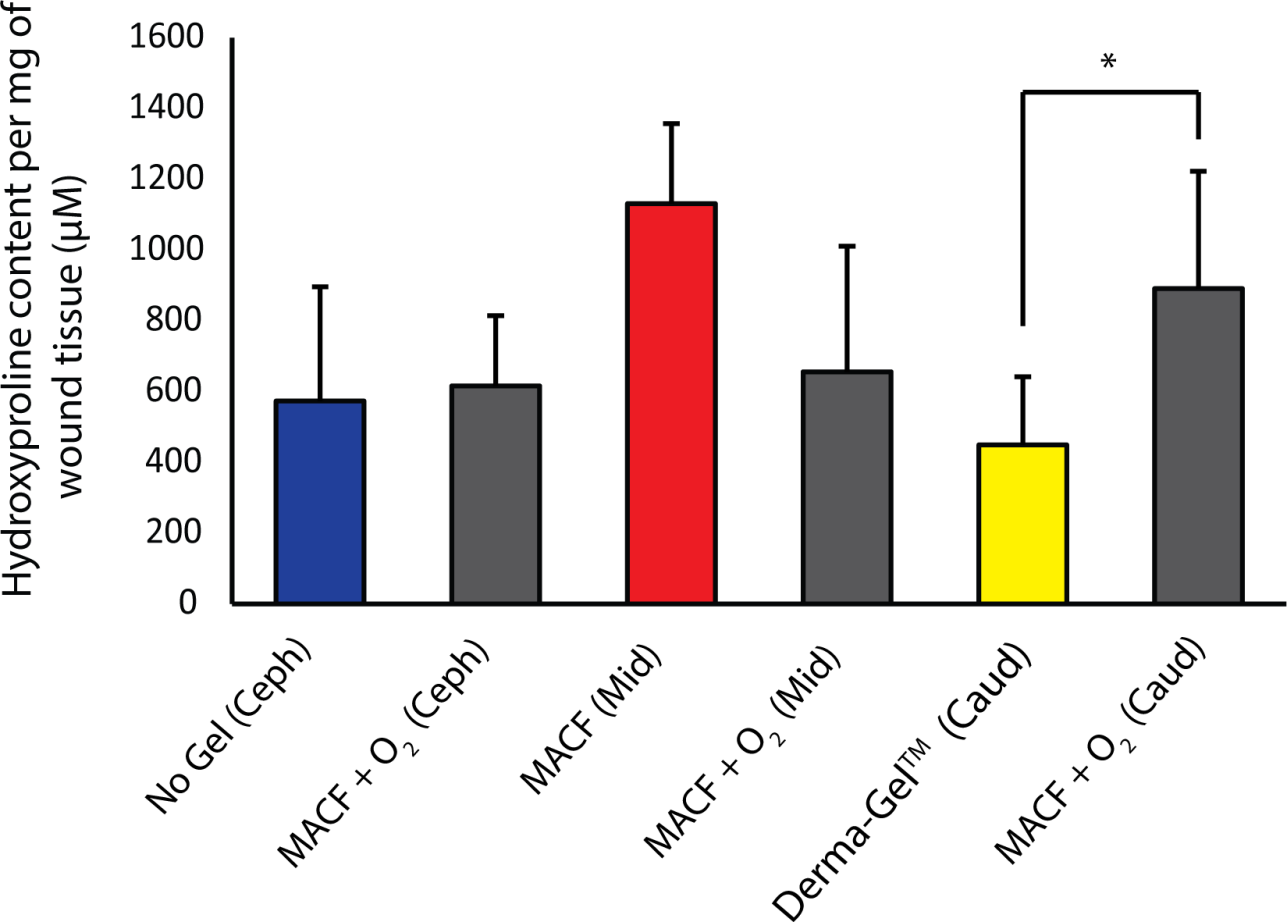
Total hydroxyproline content per 1 mg of digested wound tissue measured by hydroxyproline assay at day 21. * denotes significance by ANOVA with t-test within regions, (*p* < 0.05). Mean+/-SD, *n* = 3 (pigs), s = 2 (wounds/region).

Hydroxyproline assays were performed on hydrolyzed wound tissue extracts to infer the amount of hydroxyproline integrated into the wound during the repair process (Fig 4). Statistically significant differences were found in regions when MACF + O_2_ was compared among regions (*p* < 0.05); hence, samples were only compared within the same regions with n = 3 (pigs) and s = 2 (wounds/region). It was found that in the caudal region, the total hydroxyproline content in MACF + O_2_ treated wounds (891 ± 332 μM/mg of wound tissue) was significantly higher when compared to Derma-Gel treated wounds (450 ± 191 μM/mg) (*p* < 0.05). Comparisons in the cephalad and middle regions did not reveal statistically significant differences (*p* > 0.05).

### Hematoxylin and eosin staining: Length of epithelial tongue

**Fig 5:**
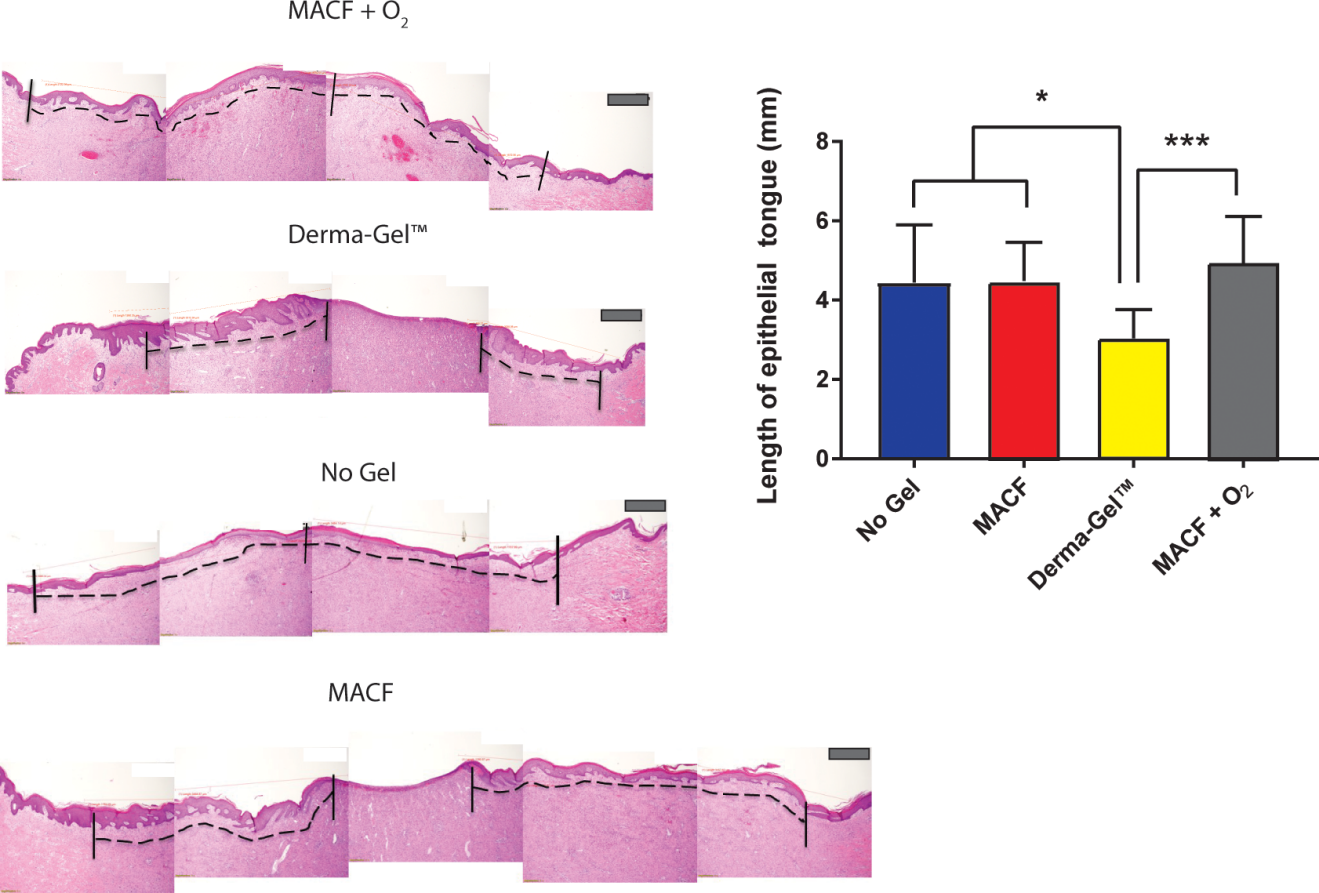
Hematoxylin and Eosin staining of wound tissue sections showing re-epithelialization. * denotes significance by one factor ANOVA with Tukey’s post hoc, (*p* < 0.05) and *** denotes significance by one factor ANOVA, (*p* < 0.01). Mean+/-SD, *n* = 3 (pigs), s = 4 (wounds/region). The dotted line on the representative images demonstrates the length of epithelial layer from the edge of the wound to the tip of epithelial tongue. Longer epithelial tongue shows better closure and more migration of keratinocytes. Scale bar is 200 μm.

H&E stained wound sections were imaged and analyzed to measure the length of the resulting epithelial tongues at the endpoint (Fig 5). Before analyses, MACF + O_2_ results in all regions (cephalad, middle and caudal) were compared and were not found to be significantly different (p > 0.05) by one-way ANOVA. Therefore, data from all the MACF + O_2_ treated wounds was combined for statistical comparison with other treatment groups to yield n = 3 (pigs) and s = 4 (wounds/region). Derma-Gel treated wounds had shorter epithelial tongues (3.04 ± 0.73 mm) as compared to MACF + O_2_ (4.59 ± 1.12 mm) (*p* < 0.0001). Lengths of epithelial tongues for MACF (4.48 ± 0.97 mm) and No Gel (4.44 ± 1.45 mm) were similar to MACF + O_2_ (*p* = 0.65 and *p* = 0.59 respectively). Furthermore, the length of epithelial tongues for Derma-Gel treated wounds were found to be significantly lower than No Gel (*p* = 0.04) and MACF treatments (*p* = 0.03).

### PicroSirius Red staining: Alignment of collagen fibers

**Fig 6:**
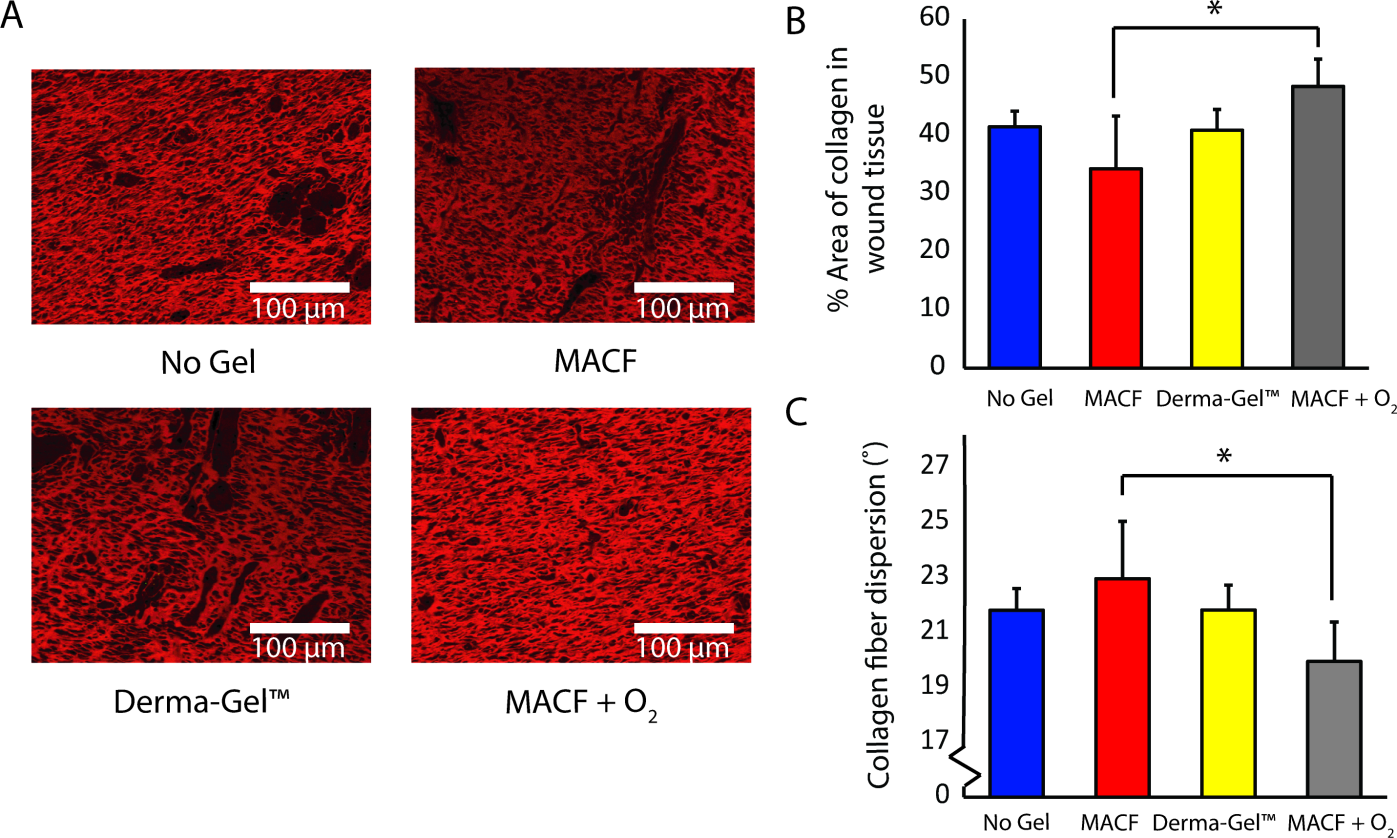
Picrosirius Red staining on wound tissue sections shows uniformly organized collagen deposition. A) Picrosirius red stained tissue images. B) % Area of collagen in tissue sample. C) Dispersion of collagen fiber orientation/directionality. Letters denote significance by ANOVA, (*p* < 0.05); n = 3 (wounds/treatment) for both collagen area and dispersion.

Picrosirius Red staining (Fig 6) revealed that MACF + O_2_ treated wounds (48.48 ± 4.68%) possessed more % area of collagen as compared to MACF treated dressings (34.30 ± 9.09%, *p* < 0.05). Further, MACF + O_2_ treated wounds showed a trend of higher collagen area % compared to No Gel (41.46 ± 2.68%) and Derma-Gel (40.92 ± 3.56%) controls. The similar trend was seen in terms of collagen fiber dispersion where MACF + O_2_ treated wounds (19.93 ± 1.43°) were significantly less dispersed than MACF (22.94 ± 2.0°, *p* < 0.05; Fig 6C) but not-significant but lower than No Gel (21.79 ± 0.78°) and Derma-Gel (21.80 ± 0.91°).

### H&E and Von Willebrand factor immunostaining: Neovascularization

**Fig 7:**
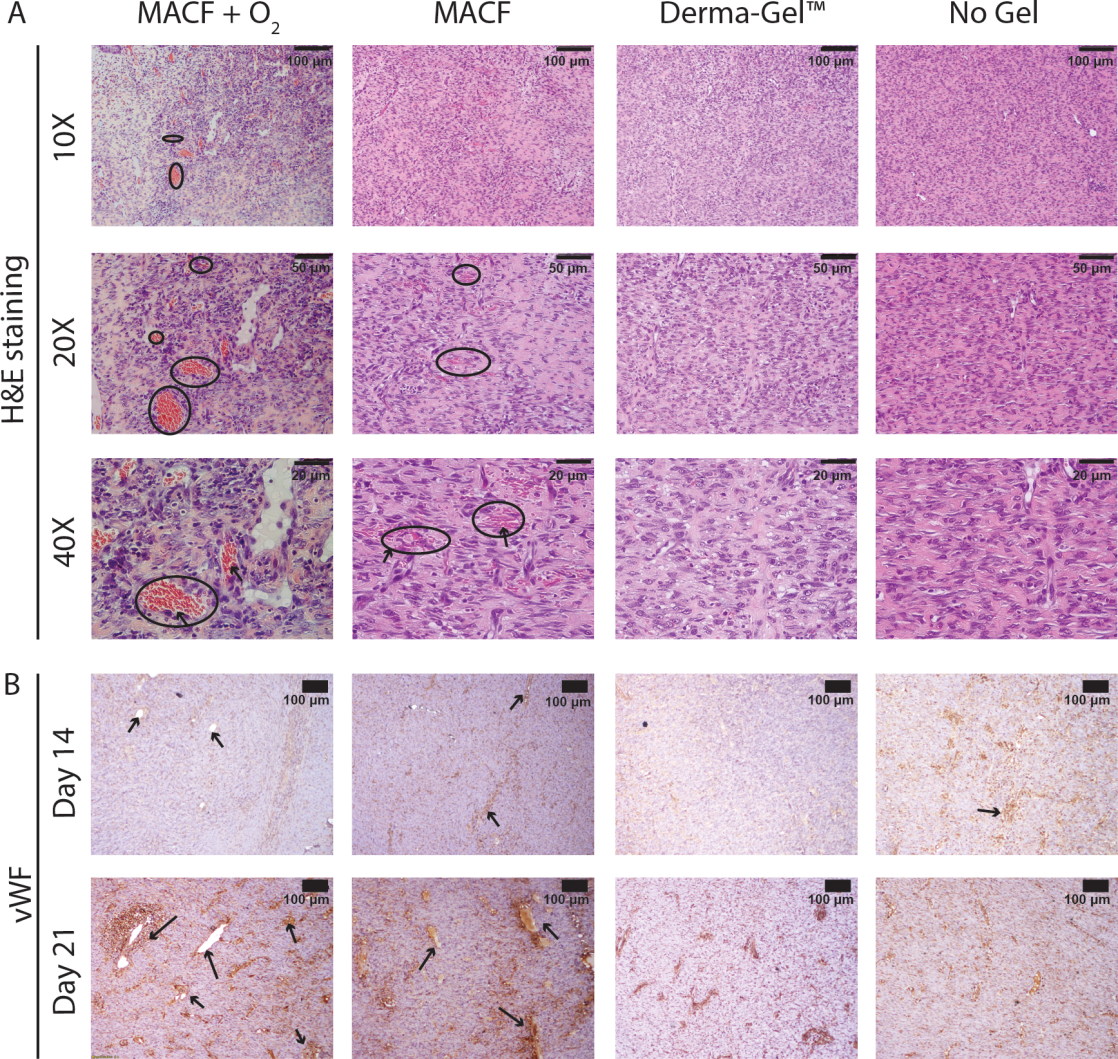
Oxygenated hydrogel treatments may help sustain neovascularization. A) H&E staining reveals circulating blood cells (arrows) in capillaries (circled). B) Von Willebrand factor immunostaining on wound tissue sections to mark (arrows) newly formed capillaries. Representative images for all wound regions.

H&E stained sections showed that oxygenating treatments (MACF + O_2_ and MACF) showed more developed capillaries with presence on circulating red blood cells (RBCs, Fig 7A). Conversely, Derma-Gel and No Gel controls only showed initial sprouting with minimum presence or absence of RBCs. Von Willebrand factor staining (Fig 7B) from day 14, confirmed that all groups except Derma-Gel treated wounds showed signs of angiogenesis with small capillaries and blood vessels. At day 21, these capillaries and blood vessels developed more prominently and were noticeably larger in diameter in MACF + O_2_ treated wounds as compared to Derma-Gel and No Gel in their corresponding regions.

### Cytokeratin immunostaining: Cytokeratins

**Fig 8:**
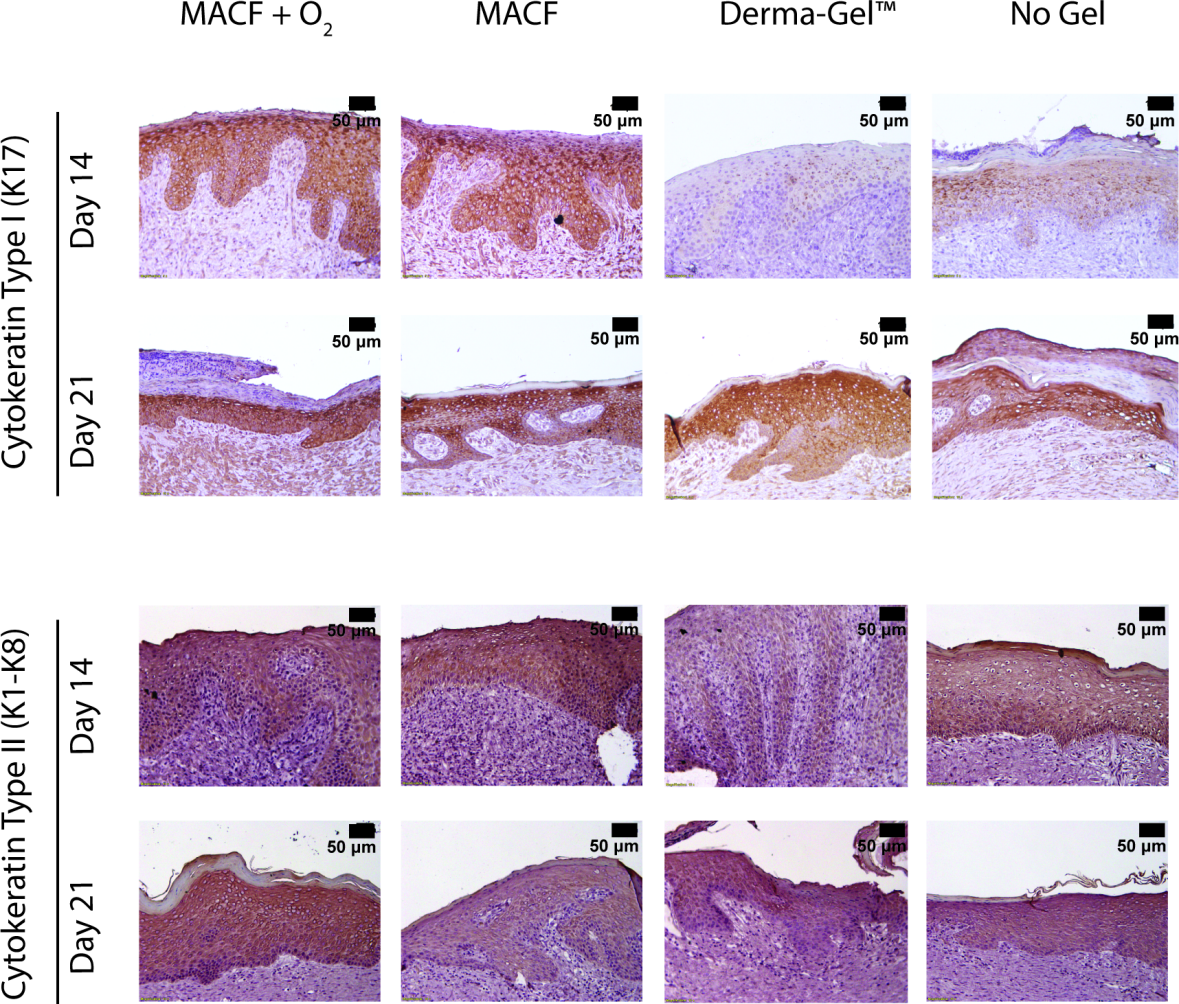
Cytokeratins Type I and II immunostaining shows MACF + O_2_ dressings may improve regulation of both cytokeratins suggesting regenerative epithelialization by the maturation of keratinocytes in later stages. Stained cells are dark brown in color. Representative images for all wound regions.

Cytokeratin antibodies were used to stain for cytokeratin type II and I. Cytokeratin type I positive staining was seen earlier at day 14 in both MACF hydrogel dressings (+ O_2_ and none) treated wounds (Fig 8). By day 21, all of the wounds demonstrated a similar abundance of cytokeratin I. At day 21, cytokeratins were prevalent in the bottom layers of epidermis and papillary region contrary to day 14, where it is throughout all of the epidermal layers. Cytokeratin II was present in MACF + O_2_, MACF, and non-treated wounds (Fig 8). Derma-Gel had little to no early expression of both cytokeratins II and I on day 14.

## Discussion

In this study, novel oxygenating hydrogel dressings, MACF + O_2_, were evaluated in an acute porcine wound model and compared to control treatments: MACF, Derma-Gel, and No Gel, all covered with secondarily wetted Allevyn foam dressing. Our previous work demonstrated that oxygenated hydrogel dressings sustain additional local oxygen via a simple sheet hydrogel [9].

Wound closure results (Fig 2) show that MACF + O_2_ treated wounds closed faster as compared to Derma-Gel and MACF treated wounds (Fig 2). This outcome can most likely be attributed to the fact that MACF + O_2_ hydrogel dressings beneficially enhance local oxygenation both short and long term [20]. Additional oxygen is known to improve and sustain cell migration and proliferation processes [26]. Somewhat surprising was the result that MACF + O_2_ treated wounds closed similar to No Gel at all timepoints (Fig 2, p > 0.05). The overriding importance and influence of moisture maintenance may be the key contributor and confounding factor for this result, as moisture plays a vital role in acute wound healing and improves wound closure [27,28]. A similar excisional full thickness porcine wound healing study studying the effect of wound hydration showed that 12 days post wounding 86% of saline treated wounds completely re-epithelialized compared to 0% of dry wounds [29]. Further, it has been previously shown that the acceleration of wound closure could be deleterious for granulation and reinnervation of newly formed tissues [30,31]. Data specifically suggest that activation and proliferation of myofibroblasts and subsequent coordination of peripheral nerves regrowth are important components for successful wound healing [32]. Interestingly, accelerating wound closure alone does not necessarily help granulation tissue and nerve fiber regrowth. In fact, accelerated wound closure in chronic critical defect wounds accelerated wound closure without proper granulation bed formation can cause infection, necrotic tissue formation and scarring further hurting preventing skin reinnervation and granulation tissue formation. Growing evidence has also demonstrated that the skin innervation influences wound healing as several neuromodulators mediate and improve wound healing processes [33,34], suggesting that, cutaneous nerve regrowth is vital for successful wound regeneration. Thus, acceleration of wound closure alone may not be beneficial for skin innervation and wound remodeling. It is vital to focus on cellular proliferative processes and skin innervation while determining the quality of wound healing. In future studies, we can specially study these mechanisms including the potential effects of MACF treatments on reinnervation of wounds. In the present study, although we did not see differences in MACF + O_2_ and No Gel in wound closure and re-epithelialization, substantial differences were observed regarding quality and maturity of newly formed wound tissue between MACF + O_2_ and No Gel and between MACF + O_2_ and Derma-Gel as supported by biochemical assays (Figs 3 and 4) and histology/IHC results (Figs 5–8).

Our previous study [20] in an acute rat model revealed that MACF + O_2_ dressings improved collagen synthesis. The present study suggests similar findings in porcine wound healing as demonstrated by probing free and total hydroxyproline content in wounds on days 14 and 21 (Figs 3 and 4). Hydroxyproline is vital to wound healing and collagen formation, and it forms through arginine and proline metabolism in the presence of sufficient oxygen [35]. Importantly, free hydroxyproline was significantly upregulated due to MACF + O_2_ as compared to No Gel at days 14 and 21 and compared to Derma-Gel at day 14 (*p* < 0.05, Fig 3), further MACF + O_2_ resulted in the highest means in each region at both time points. These results support the finding that MACF upregulates free hydroxyproline synthesis via arginine and proline metabolism at earlier time points in wound healing. Precursor metabolites of hydroxyproline include proline and arginine, and arginine interacts with oxygen in the presence of arginase enzyme to create proline, which later is converted into free hydroxyproline via prolyl 4-hydroxylase [20]. Total hydroxyproline from wound tissue samples is equivalent to the total collagen in the wound [36], and this measure was greatest in oxygenated MACF hydrogel dressings as compared to Derma-Gel (Fig 4, p < 0.05). Our results suggest that commercial hydrogel Derma-Gel had poor total hydroxyproline content as compared to MACF treatments in wound tissue over time. Moreover, non-oxygenated MACF also can sequester oxygen from the atmosphere and supply it to the wound to improve available hydroxyproline; thus, explaining the fact that we did not see difference between oxygenated and non-oxygenated MACF (Figs 3 and 4). Overall, enhanced hydroxyproline synthesis resulted in denser and more organized collagen fibers in MACF + O_2_ treated wounds as compared to non oxygenated MACF treatment and slightly better than other controls (Fig 6).This suggests role of oxygen in collagen synthesis as well as maturation through alignment is important and MACF + O_2_ can support this function.

Wound re-epithelialization results followed similar trends as the wound closure results. Interestingly, the Derma-Gel synthetic hydrogel exhibited the least re-epithelialization amongst all the treatments (Fig 5, p < 0.0001). This can primarily be due to the excess moisture and oxygen provided by MACF + O_2_ hydrogel dressing. The difference between MACF, No Gel (wet foam dressing) and MACF + O_2_ was not significant (*p* = 0.65 and 0.59 respectively). Technically, all treatments provided moisture to the wound due to the secondary, wetted foam dressing and, as previously discussed, moisture plays an important role in re-epithelialization.

Another important aspect of wound repair is the formation of the new vascular networks [37] to support the healing process and the final tissues that form. In our study, large and more developed blood vessels and capillaries were seen in MACFs as compared to Derma-Gel and No Gel treated wounds (Fig 7). MACF + O_2_ treated wounds show similar vascularization than MACF treated wounds alone. Oxygen regulates angiogenesis via cellular oxygen sensing, which results in the formation of vascular networks and microvasculature [38]. It has been previously reported that *in vitro* chronic hypoxia (<1% O_2_) inhibits proliferation of endothelial cells and limits the potential of vascular smooth muscles cells to dedifferentiate into a proliferative phenotype [39]. Topical, localized oxygenation can ensure tissue doesn’t suffer from chronic hypoxia. At the same time, a lack of preexisting vascular networks may induce acute tissue hypoxia (5% O_2_), which can sustain the angiogenesis process [40].

Looking at keratinocyte-specific markers, cytokeratin I and II were both strikingly upregulated in MACF hydrogel dressing treatments as compared to Derma-Gel and No Gel (Fig 8). Further, results at day 21 demonstrated accelerated epithelial maturation due to treatment with MACF. These results further support a role for oxygen availability in keratinocyte differentiation, which is known specifically to be heavily dependent on energy synthesis [41,42]. Oxygen availability affects keratinocyte differentiation through mitochondrial reactive oxygen species mechanism [43] and improves differentiation to yield cytokeratin I and II expressing cells [44]. These results are supported by *in vitro* work that has shown that in low oxygen tensions (< 21%) keratinocyte differentiation is reduced as compared to responses in atmospheric oxygen [41]. Overall, results suggest that MACF + O_2_ hydrogel dressings supported differentiation and maturation of keratinocytes, which was accelerated as compared to all other treatments.

A major limitation of this study is the lack of quantitative oxygen concentration data in wounds in live animals. Although application of MACF dressing (with or without oxygen) has shown to improve neovascularization and epithelial maturation, the fact that this might be due to other components of MACF or a combination of both oxygen and MACF components cannot be discounted. Lack of data presenting direct wound oxygen enrichment makes the speculation broader. Thus, additional proof is needed to justify that these improvements are directly due to enhanced local oxygenation and not the other MACF components. Oxygen measurements using phosphorescence-based commercial dissolved oxygen sensors were attempted during this study but did not yield useful data mainly because of the slow response times and boundary layer issues associated with the method. A more reliable system needs to be employed in the future to capture *in-situ* oxygen concentration changes in dressings and wounds to connect to the observed healing responses. One potential method could be electron paramagnetic resonance (EPR) oximetry, but would require a specialized EPR setup and chamber to image animals [45].

## Conclusions

In this study, treatment with MACF hydrogel dressings in general enhanced wound healing in an acute porcine wound model as evidenced by improved collagen synthesis, better neovascularization, and accelerated keratinocyte maturation when compared to No Gel and commercially available Derma-Gel dressings. However, no distinct benefit was observed in terms of wound closure and reepithelialization. Additionally, biochemical analyses confirmed hydroxyproline synthesis and uptake in collagen synthesis dependence on available oxygen, suggested that additional localized oxygen can potentially contribute to successful and enhanced wound healing via improved collagen synthesis and organization. Furthermore, it appears an overabundance of oxygen is not necessarily needed for these benefits, as results from MACF and MACF + O_2_ were similar. The combined results (histology, LC-MS/MS, and biochemistry) of this *in vivo* porcine wound healing study confirm beneficial oxygenating MACF + O_2_ results in a higher animal wound model as compared to our previous rodent study [20].

## Supporting information

S1 Table Gilman parameter data set used for wound closure analysis (Fig 2). (DOCX)

S2 Table Epithelial tongue length data calculated by image analysis (Fig 5). (DOCX)

S3 Table Percent collagen area in wound tissue calculated by image processing in Image J (Fig 6B). (DOCX)

S4 Table Dispersion in alignment of collagen fibers in wound tissue (Fig 6C). (DOCX)

S5 Table Free hydroxyproline concentrations from LC-MS/MS analysis (Fig 3). (DOCX)

S6 Table Total hydroxyproline concentration from biochemical assay (Fig 4). (DOCX)
