# Supplementary material for: Fluorinated methacrylamide chitosan hydrogel dressings enhance healing in an acute porcine wound model
Source: PLoS One. 2018 Sep 5;13(9):e0203371. doi: 10.1371/journal.pone.0203371 (PMC6124756; doi:10.1371/journal.pone.0203371)
Supplement: S1 Table — (DOCX) [file pone.0203371.s001.docx]

S1 Table: Gilman parameter data set used for wound closure analysis (Fig 2).

| **Obs** | **Pig** | **Trt** | **Day2** | **Day7** | **Day9** | **Day12** | **Day14** | **Day17** | **Day21** |
| --- | --- | --- | --- | --- | --- | --- | --- | --- | --- |
| **1** | 3 | No Gel | -0.12 | -0.12 | 0.140 | 0.22 | 0.34 | 0.54 | 0.68 |
| **2** | 3 | No Gel | -0.02 | 0.03 | 0.160 | 0.33 | 0.44 | 0.64 | 0.83 |
| **3** | 3 | No Gel | 0.30 | 0.22 | 0.370 | 0.52 | 0.55 | 0.68 | 0.88 |
| **4** | 3 | No Gel | -0.01 | 0.07 | 0.250 | 0.35 | 0.43 | 0.54 | 0.83 |
| **5** | 4 | No Gel | -0.07 | 0.00 | 0.140 | 0.30 | 0.42 | 0.57 | 0.89 |
| **6** | 4 | No Gel | 0.08 | 0.11 | 0.290 | 0.49 | 0.57 | 0.74 | 1.04 |
| **7** | 4 | No Gel | 0.01 | 0.17 | 0.390 | 0.56 | 0.66 | 0.77 | 1.06 |
| **8** | 4 | No Gel | 0.05 | 0.23 | 0.310 | 0.54 | 0.60 | 0.68 | 1.01 |
| **9** | 6 | MACF+O2 | -0.08 | -0.12 | 0.120 | 0.21 | 0.67 | 0.97 | 1.12 |
| **10** | 6 | MACF+O2 | -0.03 | 0.05 | 0.270 | 0.48 | 0.76 | 1.25 | 1.28 |
| **11** | 6 | MACF+O2 | 0.01 | -0.05 | 0.100 | 0.26 | 0.47 | 0.86 | 1.03 |
| **12** | 6 | MACF+O2 | -0.90 | -0.92 | -0.082 | -0.66 | -0.44 | -0.09 | 1.04 |
| **13** | 3 | MACF+O2 | -0.01 | -0.10 | 0.210 | 0.34 | 0.58 | 0.66 | 0.91 |
| **14** | 3 | MACF+O2 | 0.03 | -0.04 | 0.270 | 0.40 | 0.58 | 0.58 | 0.89 |
| **15** | 3 | MACF+O2 | 0.09 | 0.14 | 0.180 | 0.28 | 0.45 | 0.61 | 0.90 |
| **16** | 3 | MACF+O2 | 0.03 | 0.12 | 0.270 | 0.40 | 0.46 | 0.55 | 0.82 |
| **17** | 4 | MACF+O2 | 0.02 | -0.05 | 0.200 | 0.49 | 0.57 | 0.68 | 0.99 |
| **18** | 4 | MACF+O2 | 0.04 | -0.01 | 0.200 | 0.44 | 0.49 | 0.65 | 0.94 |
| **19** | 4 | MACF+O2 | -0.02 | 0.08 | 0.230 | 0.56 | 0.58 | 0.66 | 0.82 |
| **20** | 4 | MACF+O2 | -0.02 | 0.05 | 0.280 | 0.46 | 0.53 | 0.52 | 0.72 |
| **21** | 6 | No Gel | -0.07 | 0.01 | 0.130 | 0.39 | 0.90 | 1.02 | 1.32 |
| **22** | 6 | No Gel | -0.08 | 0.02 | 0.090 | 0.25 | 0.65 | 1.31 | 0.89 |
| **23** | 6 | No Gel | -0.03 | 0.14 | 0.280 | 0.47 | 0.98 | 1.35 | 1.05 |
| **24** | 6 | No Gel | -0.05 | 0.12 | 0.200 | 0.41 | 0.97 | 1.15 | 1.33 |
| **25** | 3 | MACFatm | -0.02 | -0.03 | 0.150 | 0.27 | 0.35 | 0.37 | 0.69 |
| **26** | 3 | MACFatm | 0.07 | 0.06 | 0.230 | 0.40 | 0.48 | 0.56 | 0.90 |
| **27** | 3 | MACFatm | 0.08 | 0.18 | 0.300 | 0.51 | 0.51 | 0.55 | 0.88 |
| **28** | 3 | MACFatm | 0.10 | 0.07 | 0.210 | 0.41 | 0.41 | 0.57 | 0.80 |
| **29** | 4 | MACFatm | -0.66 | -0.51 | -0.310 | -0.20 | 0.00 | 0.07 | 0.19 |
| **30** | 4 | MACFatm | -0.50 | -0.42 | -0.260 | . | 0.00 | 0.11 | 0.45 |
| **31** | 4 | MACFatm | -0.61 | -0.51 | -0.390 | -0.10 | 0.00 | 0.08 | 0.43 |
| **32** | 4 | MACFatm | -0.61 | -0.48 | -0.450 | -0.16 | 0.00 | 0.08 | 0.38 |
| **33** | 6 | MACF+O2 | -0.02 | 0.03 | 0.240 | 0.43 | 0.66 | 1.03 | 1.30 |
| **34** | 6 | MACF+O2 | 0.10 | 0.21 | 0.350 | 0.44 | 0.77 | 1.02 | 1.10 |
| **35** | 6 | MACF+O2 | -0.01 | -0.01 | 0.220 | 0.36 | 0.64 | 1.02 | 1.13 |
| **36** | 6 | MACF+O2 | 0.05 | 0.01 | 0.150 | 0.24 | 0.58 | 0.81 | 1.22 |
| **37** | 3 | MACF+O2 | 0.12 | 0.12 | 0.170 | 0.40 | 0.42 | 0.58 | 0.88 |
| **38** | 3 | MACF+O2 | 0.15 | 0.09 | 0.190 | 0.41 | 0.45 | 0.59 | 0.89 |
| **39** | 3 | MACF+O2 | 0.02 | -0.07 | 0.090 | 0.31 | 0.43 | 0.50 | 0.72 |
| **40** | 3 | MACF+O2 | -0.20 | -0.08 | 0.050 | 0.40 | 0.46 | 0.53 | 0.91 |
| **41** | 4 | MACF+O2 | 0.01 | 0.01 | 0.150 | 0.50 | 0.46 | 0.67 | 0.87 |
| **42** | 4 | MACF+O2 | -0.04 | -0.11 | 0.070 | 0.39 | 0.50 | 0.66 | 1.04 |
| **43** | 4 | MACF+O2 | -0.03 | 0.09 | 0.310 | 0.56 | 0.57 | 0.72 | 0.90 |
| **44** | 4 | MACF+O2 | -0.05 | 0.05 | 0.210 | 0.44 | 0.58 | 0.74 | 1.02 |
| **45** | 6 | MACFatm | -0.07 | -0.01 | 0.130 | 0.28 | 0.67 | 0.89 | 1.16 |
| **46** | 6 | MACFatm | -0.05 | 0.02 | 0.190 | 0.30 | 0.73 | 0.92 | 1.32 |
| **47** | 6 | MACFatm | 0.12 | 0.16 | 0.340 | 0.70 | 0.96 | 0.01 | 1.26 |
| **48** | 6 | MACFatm | -0.02 | 0.24 | 0.370 | 0.72 | 0.96 | 0.91 | 1.29 |
| **49** | 3 | Derma-Gel | -0.17 | -0.18 | 0.030 | 0.27 | 0.33 | 0.30 | 0.66 |
| **50** | 3 | Derma-Gel | -0.11 | -0.20 | -0.080 | 0.18 | 0.27 | 0.19 | 0.57 |
| **51** | 3 | Derma-Gel | -0.66 | -0.74 | -0.590 | -0.40 | -0.28 | -0.31 | -0.11 |
| **52** | 3 | Derma-Gel | -0.15 | -0.09 | 0.070 | 0.28 | 0.33 | 0.37 | 0.56 |
| **53** | 4 | Derma-Gel | -0.25 | -0.09 | 0.060 | 0.25 | 0.30 | 0.40 | 0.81 |
| **54** | 4 | Derma-Gel | -0.15 | -0.15 | 0.040 | 0.32 | 0.34 | 0.41 | 0.90 |
| **55** | 4 | Derma-Gel | -1.06 | -1.04 | -1.000 | -0.77 | -0.66 | -0.50 | -0.26 |
| **56** | 4 | Derma-Gel | -0.96 | -0.81 | -0.830 | -0.73 | -0.51 | -0.47 | -0.63 |
| **57** | 6 | Derma-Gel | -0.20 | -0.17 | 0.060 | 0.14 | 0.51 | 0.80 | 1.18 |
| **58** | 6 | Derma-Gel | 0.02 | -0.07 | 0.030 | 0.23 | 0.62 | 0.87 | 1.18 |
| **59** | 6 | Derma-Gel | -0.99 | -0.08 | 0.090 | 0.25 | 0.54 | 0.96 | 1.23 |
| **60** | 6 | Derma-Gel | -0.07 | -0.09 | 0.150 | 0.35 | 0.58 | 0.86 | 1.19 |
| **61** | 3 | MACF+O2 | 0.05 | 0.03 | 0.200 | 0.36 | 0.53 | 0.64 | 0.80 |
| **62** | 3 | MACF+O2 | 0.17 | 0.10 | 0.190 | 0.45 | 0.51 | 0.63 | 0.87 |
| **63** | 3 | MACF+O2 | -0.11 | -0.01 | 0.150 | 0.26 | 0.42 | 0.41 | 0.75 |
| **64** | 3 | MACF+O2 | -0.03 | 0.06 | 0.170 | 0.40 | 0.48 | 0.57 | 0.81 |
| **65** | 4 | MACF+O2 | -0.15 | -0.10 | 0.070 | 0.27 | 0.32 | 0.52 | 0.92 |
| **66** | 4 | MACF+O2 | -0.29 | -0.35 | -0.110 | 0.21 | 0.29 | 0.52 | 0.83 |
| **67** | 4 | MACF+O2 | -0.18 | 0.00 | 0.140 | 0.45 | 0.48 | 0.61 | 0.73 |
| **68** | 4 | MACF+O2 | -0.13 | 0.06 | 0.220 | 0.58 | 0.55 | 0.61 | 0.70 |
| **69** | 6 | MACF+O2 | -0.04 | -0.16 | 0.010 | 0.15 | 0.59 | 0.78 | 1.32 |
| **70** | 6 | MACF+O2 | 1.57 | 1.57 | 1.660 | 1.76 | 1.93 | 1.99 | 2.12 |
| **71** | 6 | MACF+O2 | -0.12 | -0.13 | 0.050 | 0.29 | 0.54 | 0.76 | 1.18 |
| **72** | 6 | MACF+O2 | -0.11 | -0.05 | 0.090 | 0.26 | 0.54 | 0.83 | 1.16 |
