# Supplementary material for: Fluorinated methacrylamide chitosan hydrogel dressings enhance healing in an acute porcine wound model
Source: PLoS One. 2018 Sep 5;13(9):e0203371. doi: 10.1371/journal.pone.0203371 (PMC6124756; doi:10.1371/journal.pone.0203371)
Supplement: S2 Table — (DOCX) [file pone.0203371.s002.docx]

S2 Table: Epithelial tongue length data calculated by image analysis (Fig 5).

| Treatment | Length of epithelial tongue in µm |
| --- | --- |
| No Gel | 2521.97 |
| No Gel | 2989.27 |
| No Gel | 2954.08 |
| No Gel | 2695.60 |
| No Gel | 5674.29 |
| No Gel | 5538.41 |
| No Gel | 5975.19 |
| No Gel | 4981.02 |
| No Gel | 5681.86 |
| No Gel | 5423.59 |
| MACF+O_2_ | 3382.86 |
| MACF+O_2_ | 3471.10 |
| MACF+O_2_ | 5584.17 |
| MACF+O_2_ | 3062.82 |
| MACF+O_2_ | 6277.41 |
| MACF+O_2_ | 4324.35 |
| MACF+O_2_ | 4258.14 |
| MACF+O_2_ | 5744.77 |
| MACF+O_2_ | 5311.25 |
| MACF+O_2_ | 6229.15 |
| MACFatm | 3011.68 |
| MACFatm | 4553.41 |
| MACFatm | 3407.49 |
| MACFatm | 4459.55 |
| MACFatm | 4698.41 |
| MACFatm | 5204.55 |
| MACFatm | 3290.10 |
| MACFatm | 5125.77 |
| MACFatm | 5045.88 |
| MACFatm | 6051.59 |
| MACF+O_2_ | 4579.54 |
| MACF+O_2_ | 4649.58 |
| MACF+O_2_ | 4804.37 |
| MACF+O_2_ | 7264.62 |
| MACF+O_2_ | 5041.82 |
| MACF+O_2_ | 5047.33 |
| MACF+O_2_ | 5509.91 |
| MACF+O_2_ | 6999.43 |
| MACF+O_2_ | 7393.32 |
| MACF+O_2_ | 4430.74 |
| Derma-Gel | 3552.94 |
| Derma-Gel | 2209.11 |
| Derma-Gel | 2519.27 |
| Derma-Gel | 2317.52 |
| Derma-Gel | 3711.81 |
| Derma-Gel | 3585.09 |
| Derma-Gel | 2418.04 |
| Derma-Gel | 2317.56 |
| Derma-Gel | 3880.42 |
| Derma-Gel | 3847.62 |
| MACF+O_2_ | 4259.37 |
| MACF+O_2_ | 5958.68 |
| MACF+O_2_ | 3539.83 |
| MACF+O_2_ | 3879.64 |
| MACF+O_2_ | 7065.37 |
| MACF+O_2_ | 4781.02 |
| MACF+O_2_ | 4589.71 |
| MACF+O_2_ | 3609.52 |
| MACF+O_2_ | 3786.67 |
| MACF+O_2_ | 4380.68 |
