# Supplementary material for: Fluorinated methacrylamide chitosan hydrogel dressings enhance healing in an acute porcine wound model
Source: PLoS One. 2018 Sep 5;13(9):e0203371. doi: 10.1371/journal.pone.0203371 (PMC6124756; doi:10.1371/journal.pone.0203371)
Supplement: S3 Table — (DOCX) [file pone.0203371.s003.docx]

S3 Table: % Collagen area in wound tissue calculated by Image processing in Image J (Fig 6B).

| Treatment | Collagen Area (pixel^2^) from Image J | Total Area (pixel^2^) from Image J | % collagen density |
| --- | --- | --- | --- |
| No Gel | 323657 | 786432 | 41.15 |
| No Gel | 348842 | 786432 | 44.35 |
| No Gel | 307021 | 786432 | 39.03 |
| MACF | 339665 | 786432 | 43.19 |
| MACF | 196758 | 786432 | 25.01 |
| MACF | 272938 | 786432 | 34.70 |
| Derma-Gel | 293181 | 786432 | 37.27 |
| Derma-Gel | 349271 | 786432 | 44.41 |
| Derma-Gel | 323044 | 786432 | 41.07 |
| MACF + O2 | 409833 | 786432 | 52.11 |
| MACF + O2 | 411801 | 786432 | 52.36 |
| MACF + O2 | 343624 | 786432 | 43.69 |
| MACF + O2 | 419853 | 786432 | 53.38 |
| MACF + O2 | 376524 | 786432 | 47.87 |
| MACF + O2 | 328018 | 786432 | 41.70 |
| MACF + O2 | 348522 | 786432 | 44.31 |
| MACF + O2 | 411209 | 786432 | 52.28 |
