# Supplementary material for: Fluorinated methacrylamide chitosan hydrogel dressings enhance healing in an acute porcine wound model
Source: PLoS One. 2018 Sep 5;13(9):e0203371. doi: 10.1371/journal.pone.0203371 (PMC6124756; doi:10.1371/journal.pone.0203371)
Supplement: S4 Table — (DOCX) [file pone.0203371.s004.docx]

S4 Table: Dispersion in alignment of collagen fibers in wound tissue (Fig 6C).

| Treatment | Direction (˚) | Diespersion (˚) | Goodness |
| --- | --- | --- | --- |
| No Gel | -11.11 | 20.9 | 1 |
| No Gel | -4.03 | 22.13 | 0.99 |
| No Gel | 11.91 | 22.35 | 1 |
| MACF | -0.4 | 20.54 | 0.99 |
| MACF | -19.22 | 24.26 | 0.99 |
| MACF | -10.8 | 24.03 | 1 |
| Derma-Gel | 3.71 | 21.34 | 1 |
| Derma-Gel | -7.78 | 22.85 | 0.99 |
| Derma-Gel | 6.2 | 21.21 | 0.99 |
| MACF + O2 | 8.37 | 20.15 | 0.99 |
| MACF + O2 | -6.49 | 19.62 | 0.99 |
| MACF + O2 | 1.78 | 20.27 | 1 |
| MACF + O2 | 0.69 | 21.94 | 0.99 |
| MACF + O2 | -7.21 | 18.13 | 0.99 |
| MACF + O2 | -0.5 | 18.19 | 0.99 |
