# Supplementary material for: Fluorinated methacrylamide chitosan hydrogel dressings enhance healing in an acute porcine wound model
Source: PLoS One. 2018 Sep 5;13(9):e0203371. doi: 10.1371/journal.pone.0203371 (PMC6124756; doi:10.1371/journal.pone.0203371)
Supplement: S5 Table — (DOCX) [file pone.0203371.s005.docx]

S5 Table: Free hydroxyproline concentrations from LC-MS/MS analysis (Fig 3).

|  | Free hydroxyproline concentration in µM | |
| --- | --- | --- |
|  | Day 14 | Day 21 |
| No Gel | 6.37 | 79.21 |
| No Gel | 7.73 | 107.31 |
| No Gel | 16.69 | 142.17 |
| No Gel | 22.01 | 46.37 |
| No Gel | 18.81 | 105.41 |
| No Gel | 20.67 | 77.37 |
| MACF+O2 | 43.91 | 111.91 |
| MACF+O2 | 35.21 | 145.24 |
| MACF+O2 | 119.97 | 222.51 |
| MACF+O2 | 122.87 | 199.44 |
| MACF+O2 | 44.54 | 135.31 |
| MACF+O2 | 122.81 | 116.21 |
| MACF | 75.57 | 42.44 |
| MACF | 45.17 | 68.41 |
| MACF | 26.27 | 42.44 |
| MACF | 49.67 | 14.23 |
| MACF | 32.44 | 39.31 |
| MACF | 26.37 | 54.97 |
| MACF+O2 | 94.74 | 58.04 |
| MACF+O2 | 62.07 | 59.41 |
| MACF+O2 | 45.67 | 58.04 |
| MACF+O2 | 26.24 | 23.84 |
| MACF+O2 | 41.74 | 68.61 |
| MACF+O2 | 23.01 | 19.35 |
| Derma-Gel | 37.41 | 20.77 |
| Derma-Gel | 29.74 | 28.44 |
| Derma-Gel | 33.17 | 18.55 |
| Derma-Gel | 41.84 | 35.47 |
| Derma-Gel | 66.64 | 16.39 |
| Derma-Gel | 26.37 | 31.17 |
| MACF+O2 | 99.77 | 22.04 |
| MACF+O2 | 101.74 | 43.01 |
| MACF+O2 | 103.34 | 34.47 |
| MACF+O2 | 38.17 | 49.97 |
| MACF+O2 | 104.01 | 21.47 |
| MACF+O2 | 220.21 | 39.04 |
