# Supplementary material for: Fluorinated methacrylamide chitosan hydrogel dressings enhance healing in an acute porcine wound model
Source: PLoS One. 2018 Sep 5;13(9):e0203371. doi: 10.1371/journal.pone.0203371 (PMC6124756; doi:10.1371/journal.pone.0203371)
Supplement: S6 Table — (DOCX) [file pone.0203371.s006.docx]

S6 Table: Total hydroxyproline concentration from biochemical assay (Fig 4).

| Treatments | Hydroxyproline concentration in µM |
| --- | --- |
| No Gel | 701.02 |
| No Gel | 1060.05 |
| No Gel | 389.83 |
| No Gel | 493.16 |
| No Gel | 220.33 |
| MACF+O2 | 456.16 |
| MACF+O2 | 619.80 |
| MACF+O2 | 458.04 |
| MACF+O2 | 937.03 |
| MACF+O2 | 758.66 |
| MACFatm | 469.83 |
| MACFatm | 1402.40 |
| MACFatm | 881.01 |
| MACFatm | 1209.33 |
| MACFatm | 1032.83 |
| MACF+O2 | 345.83 |
| MACF+O2 | 367.02 |
| MACF+O2 | 490.33 |
| MACF+O2 | 993.03 |
| MACF+O2 | 1083.41 |
| Derma-Gel | 551.20 |
| Derma-Gel | 182.16 |
| Derma-Gel | 375.33 |
| Derma-Gel | 692.81 |
| Derma-Gel | 448.50 |
| MACF+O2 | 892.80 |
| MACF+O2 | 1414.61 |
| MACF+O2 | 534.02 |
| MACF+O2 | 916.50 |
| MACF+O2 | 697.33 |
